# Clinical-Grade *Generation* of Active NK Cells from Cord Blood Hematopoietic Progenitor Cells for Immunotherapy Using a Closed-System Culture Process

**DOI:** 10.1371/journal.pone.0020740

**Published:** 2011-06-16

**Authors:** Jan Spanholtz, Frank Preijers, Marleen Tordoir, Carel Trilsbeek, Jos Paardekooper, Theo de Witte, Nicolaas Schaap, Harry Dolstra

**Affiliations:** 1 Department of Laboratory Medicine, Laboratory of Hematology, Radboud University Medical Centre, Nijmegen, The Netherlands; 2 Department of Hematology, Radboud University Medical Centre, Nijmegen, The Netherlands; 3 Department of Tumor Immunology, Radboud University Medical Centre, Nijmegen, The Netherlands; University of Minnesota, United States of America

## Abstract

Natural killer (NK) cell-based adoptive immunotherapy is a promising treatment approach for many cancers. However, development of protocols that provide large numbers of functional NK cells produced under GMP conditions are required to facilitate clinical studies. In this study, we translated our cytokine-based culture protocol for *ex vivo* expansion of NK cells from umbilical cord blood (UCB) hematopoietic stem cells into a fully closed, large-scale, cell culture bioprocess. We optimized enrichment of CD34^+^ cells from cryopreserved UCB units using the CliniMACS system followed by efficient expansion for 14 days in gas-permeable cell culture bags. Thereafter, expanded CD34^+^ UCB cells could be reproducibly amplified and differentiated into CD56^+^CD3^−^ NK cell products using bioreactors with a mean expansion of more than 2,000 fold and a purity of >90%. Moreover, expansion in the bioreactor yielded a clinically relevant dose of NK cells (mean: 2×10^9^ NK cells), which display high expression of activating NK receptors and cytolytic activity against K562. Finally, we established a versatile closed washing procedure resulting in optimal reduction of medium, serum and cytokines used in the cell culture process without changes in phenotype and cytotoxic activity. These results demonstrate that large numbers of UCB stem cell-derived NK cell products for adoptive immunotherapy can be produced in closed, large-scale bioreactors for the use in clinical trials.

## Introduction

Natural Killer (NK) cells are CD3^−^CD56^+^ lymphocytes that exert innate immunity against cancer and viral infections [1]. Recognition and subsequent killing of virus-infected and transformed cells by NK cells is regulated through the balance of signals from inhibitory and activating receptors [1]. Due to their strong ability to target tumor cells, NK cells have been described as promising effectors for adoptive immunotherapy against cancer [2]. It has been well demonstrated that NK cell alloreactivity can control relapse of acute myeloid leukemia (AML) without causing graft-versus-host disease (GVHD) in the setting of haploidentical stem cell transplantation (SCT) [3]. Moreover, haploidentical NK cell infusions in adult and childhood AML following lymphocyte depleting chemotherapy have provided encouraging results [4], [5]. However, only a few trials investigating adoptive NK cell infusions in patients with cancer have been conducted to date. A major obstacle is that relative small numbers of NK cells can be isolated from a regular leukapheresis products. This hampers clinical trials evaluating for NK-cell dose dependent anti-tumor responses in humans with cancer [6]–[11]. Therefore, protocols for *ex vivo* expansion and activation of NK cells are under investigation enabling clinical trials at higher NK cell dosages and to permit multiple NK cell infusions [12]–[16]. However, most protocols still deal with technical disadvantages by using supportive feeder cell lines that could lead to regulatory problems producing NK cell products for large-scale and multi-center trials. Interestingly, a recent study by Sutlu et al. reported that large amounts of highly active NK cells can be produced from peripheral blood in a closed, automated bioreactor under feeder-free conditions. [17].

Recently, we have described an alternative cytokine-based culture method with the capability of generating clinically relevant NK cell products with high cell numbers, high purity and functionality from umbilical cord blood derived hematopoietic stem cells (UCB-HSC) [18]. UCB is a very attractive source of HSC not only for allogeneic SCT, but also for producing a multitude of therapeutic cell products including NK cells [18]–[20]. An optimal procedure for the clinical-grade generation of UCB progenitor cell-derived NK cells must include a GMP-compatible HSC enrichment procedures as well as a closed-system culture system free of animal products and feeder cells. In the present study, we have investigated the feasibility of large scale NK cell generation using cryopreserved UCB units as progenitor cell source. We have optimized the enrichment of CD34^+^ cells from thawed UCB units using the CliniMACS system. Furthermore, we have evaluated CD34^+^ cells-derived NK cell generation in static cell culture bags and an automated bioreactor, with the aim of optimizing fully closed, large-scale production of highly active and functional NK cells for the use in a phase I dose-finding trial in elderly AML patients not eligible for allogeneic SCT.

## Results

### Efficient enrichment of CD34^+^ cells from cryopreserved umbilical cord blood

The overall aim of this study was to develop a closed *ex vivo* culture system for the expansion and differentiation of CD34^+^ UCB cells into NK cells followed by the subsequent log-scale generation of CD56^+^CD3^−^ NK cells. As the initiation of our culture process requires hematopoietic progenitor cells, we optimized the CD34^+^ enrichment procedure from cryopreserved UCB units using the CliniMACS system. Prior to banking in liquid nitrogen the collected UCB units used for this study (n = 16) have been reduced for red blood cells and volume using EloHAES® separation. The mean volume of 111±34 ml (range 72–175 ml) and mean WBC count of 1,503±455×10^6^ cells (range 772–2,380×10^6^) was reduced to 25 ml with a WBC count of 1,085±357×10^6^ cells (range 600–1,721×10^6^) containing 3.78±1.95×10^6^ CD34^+^ cells (range 1.73–8.72×10^6^) (Table 1). Cryopreserved UCB units were thawed and prepared for CD34^+^ selection using CliniMACS buffer containing clinical-grade DNAse. The recovery of CD34^+^ cells after thawing was 76%±16%, which resulted in a total yield of 2.79±1.59×10^6^ CD34^+^ cells (range 1.43–8.12×10^6^) for the selected UCB units (Table 1). Next, CD34^+^ cells were enriched using the CliniMACS cell separator resulting in a mean recovery of 71%±11% (range 50–91%) (Table 2). The purity of the enriched CD34^+^ product was 67%±14% (range 44–92%). Total recovery after thawing and CD34 enrichment was 53%±15% (range 33–82%) with a mean CD34^+^ cell number of 1.96×10^6^±1.27×10^6^ (range 0.89–6.34×10^6^) (Table 2). These results demonstrate that CD34^+^ cells can be efficiently enriched from volume-reduced and cryopreserved UCB units providing a clinical-grade starting product for the NK cell generation and expansion culture process.

**Table 1:** Characteristics of the UCB units after EloHAES separation and cryopreservation.

	Collected UCB		Volume reduced UCB		Thawed UCB		
	Volume	NCs	NCs	CD34^+^ cells	NCs	CD34^+^ cells	Recovery CD34^+^ cells
	ml	x10^6^	x10^6^	x10^6^	x10^6^	x10^6^	%
**Donor 1**	88	1294	790	3.90	368	2.96	76
**Donor 2**	151	1857	1312	5.88	469	3.73	63
**Donor 3**	141	1734	1378	4.96	653	3.23	65
**Donor 4**	87	1992	1588	8.72	819	8.12	93
**Donor 5**	119	1821	1106	3.68	583	2.28	62
**Donor 6**	153	1775	1519	3.17	829	2.15	68
**Donor 7**	152	1733	978	2.08	440	2.06	99
**Donor 8**	72	1210	760	2.70	403	2.07	77
**Donor 9**	78	772	600	3.96	248	1.84	46
**Donor 10**	97	927	616	1.73	386	1.69	98
**Donor 11**	81	1207	974	2.82	479	2.52	89
**Donor 12**	175	2380	1721	6.90	943	3.96	57
**Donor 13**	95	1430	1008	3.04	558	2.66	87
**Donor 14**	77	857	680	1.75	273	1.43	82
**Donor 15**	88	1223	969	2.40	563	2.14	89
**Donor 16**	130	1829	1364	2.78	821	1.82	66
**mean**	**111**	**1503**	**1085**	**3.78**	**552**	**2.79**	**76**
**SD**	**34**	**455**	**357**	**1.95**	**210**	**1.59**	**16**
**median**	**96**	**1581**	**993**	**3.11**	**518**	**2.21**	**76**
**min**	**72**	**772**	**600**	**1.73**	**248**	**1.43**	**46**
**max**	**175**	**2380**	**1721**	**8.72**	**943**	**8.12**	**99**

The table summarizes the processing of 16 UCB units used for CD34^+^ enrichment after collection, volume reduction and thawing process. Nucleated cells (NCs) were counted with the AcT10 counter (Beckman coulter). CD34^+^ cells were enumerated by single platform flow cytometry analysis. Results are depicted as mean, standard deviation, median, minimum (min) and maximum (max) volume, number of cells or percentages, respectively.

**Table 2:** Characteristics of the CD34 CliniMACS separation on thawed UCB units.

	CD34+ positive fraction			
	Recovery after CD34 enrichment only (%)	CD34^+^ cell content (%)	CD34^+^ cells (x10^6^)	Recovery of CD34^+^ cells after processing (%)
**Donor 1**	50	52	1.47	38
**Donor 2**	53	77	1.99	34
**Donor 3**	73	70	2.36	48
**Donor 4**	78	92	6.34	73
**Donor 5**	76	54	1.74	47
**Donor 6**	79	65	1.70	54
**Donor 7**	82	64	1.70	82
**Donor 8**	69	73	1.42	53
**Donor 9**	72	88	1.32	33
**Donor 10**	76	69	0.89	51
**Donor 11**	91	65	2.29	81
**Donor 12**	70	59	2.79	40
**Donor 13**	55	84	1.47	48
**Donor 14**	76	67	1.09	62
**Donor 15**	71	44	1.52	63
**Donor 16**	65	52	1.19	43
**mean**	**71**	**67**	**1.96**	**53**
**SD**	**11**	**14**	**1.27**	**15**
**median**	**73**	**66**	**1.61**	**50**
**min**	**50**	**44**	**0.89**	**33**
**max**	**91**	**92**	**6.34**	**82**

The table summarizes the results of the CD34^+^ enrichment procedure of 16 UCB units. CD34^+^ cells were enumerated by single platform flow cytometry analysis. Results are depicted as mean, standard deviation, median, minimal (min) and maximal (max) number of cells or percentages.

### Enriched CD34^+^ UCB cells can be efficiently expanded using static cell culture bags

Previously, research scale experiments in 6-well plates showed that CD34^+^ cells, enriched from frozen UCB units, can be efficiently expanded and differentiated into the NK cell lineage using our two step *ex vivo* culture process [18]. To translate this protocol into a closed culture system, we have tested *ex vivo* expansion of CD34^+^ UCB cells for two weeks in static Vuelife™ AC culture bags using CD34^+^ expansion medium I (day 0–9) and medium II (day 9–14). The mean total cell expansion for all experiments (n = 7) was 39±14 and 160±69 fold after 1 and 2 weeks of culture, respectively (data not shown). These results were similar to the rate of expansion of 192±82 (n = 7) obtained after 2 weeks in 6-well plates (data not shown), and indicate that selected CD34^+^ cells from cryopreserved UCB units can be efficiently expanded during 2 weeks of culture in disposable bags.

### Efficient expansion of highly pure NK cell products using a bioreactor

**Figure 1:**
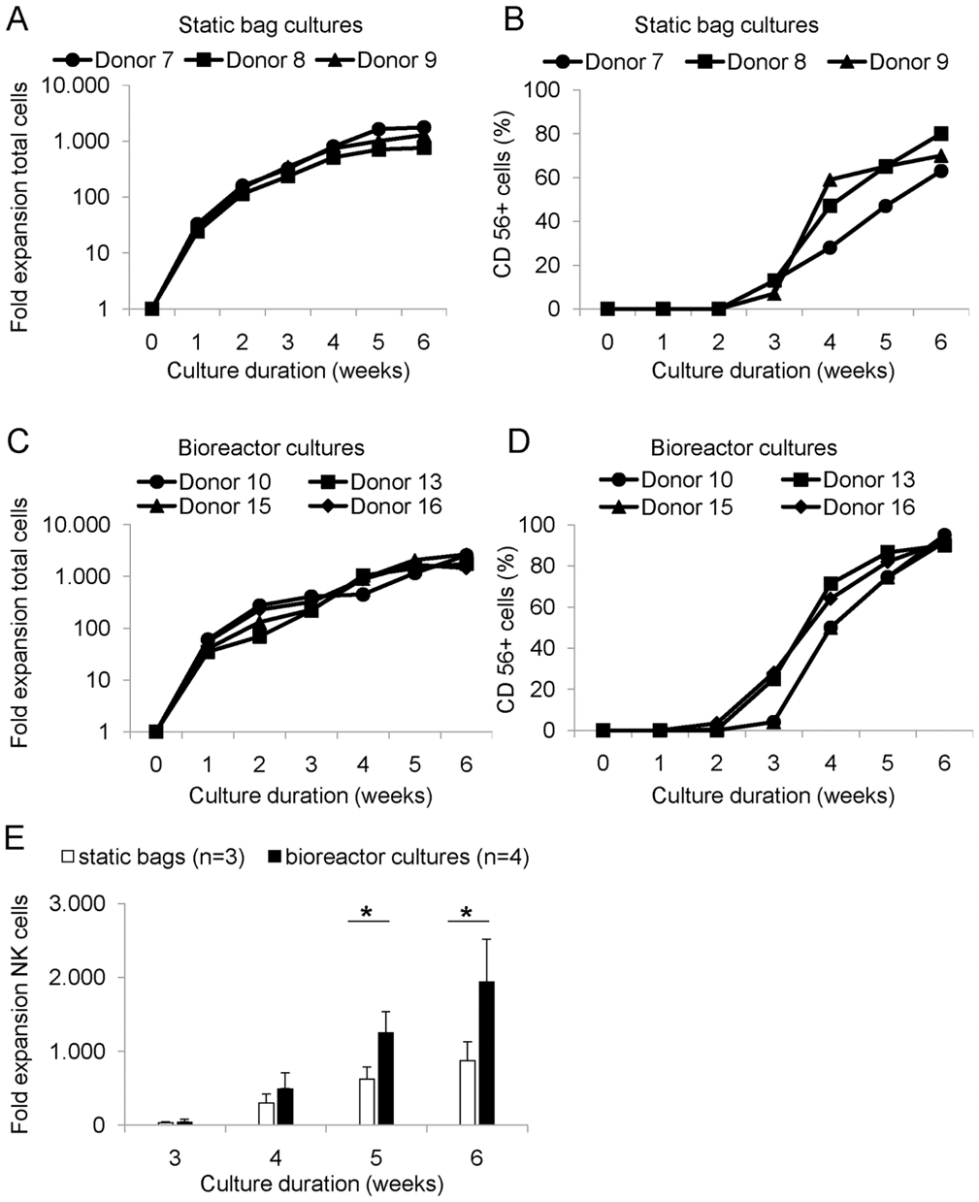
*Ex-vivo* generation of CD56^+^ NK cells from cryopreserved CD34^+^ UCB cells. CD34-enriched UCB cells were expanded for two weeks and subsequently differentiated into NK cells for four additional weeks. Cell cultures were weekly analyzed for cell numbers and phenotype using flow cytometry. (**A**) Fold expansion of total cells for each donor after initial seeding of enriched CD34^+^ UCB cells during 6 weeks of culture using static Vuelife™ cell culture bags. (**B**) CD56^+^ cell frequency for each donor during the 6 week culture period for static bag cultures. (**C**) Fold expansion of total cells for each donor after initial seeding of enriched CD34^+^ UCB cell population during 6 weeks of culture using single use bioreactors. (**D**) CD56^+^ cell frequency for each donor during the 6 week culture period for bioreactor cultures. (**E**) Mean total CD56^+^ NK cell expansion during 4 weeks of differentiation using static bag (n = 3) or bioreactor cultures (n = 4). Data are depicted as mean ± SD. The asterisk (*) represents a p-value of <0.05.

Next, we investigated whether the bag-expanded CD34^+^ UCB cells could be differentiated and further expanded into CD56^+^CD3^−^ NK cells. First, we continued the differentiation process in the same static bags as used for CD34^+^ cell expansion. Therefore, we added NK cell differentiation medium containing SCF, IL-7, IL-15 and IL-2 to the bag cultures from day 14 onward. The mean total cell expansion after 6 weeks of culture in the static bags was ∼1,300 fold (range 759–1,770; n = 3), generating NK cell products of 0.9-1.9×10^9^ CD56^+^CD3^−^ NK cells (Figure 1A and Table 3). However, *ex vivo* generation of CD56^+^CD3^−^ NK cells in bag cultures yielded in a purity of 71%±9% (Figure 1B and Table 3). The remaining non-NK cells in the product represented CD14^+^ and/or CD15^+^ mature monocytic and myelocytic cells, but no CD34^+^ cells nor CD3^+^ T cells and CD19^+^ B cells could be detected (data not shown). Because differentiation of the NK cell products was sub-optimal in the bag cultures, we next tested whether differentiation of the bag-expanded CD34^+^ cultures into the NK cell lineage could be improved using an automated bioreactor. Therefore, in a next set of experiments expanded CD34^+^ UCB cells were transferred at day 14 of culture into a bioreactor system with a minimal volume of 250 ml for starting the NK cell differentiation process. Although the mean total cell expansion at 6 weeks of culture in the bioreactor cultures, which was ∼2,100 fold (range 1,435–2,657; n = 4; Figure 3C and Table 3), was not significantly higher compared to the bag-expanded NK cells, the differentiation and expansion rate of NK cells was significantly better in the bioreactors (Figure 1D and E). Importantly, *ex vivo* generation of CD56^+^CD3^−^ NK cells in bioreactors yielded highly pure (92%±2%; n = 4) NK cell products with a total NK cell number of 1.6-3.7×10^9^ CD56^+^CD3^−^ NK cells (Table 3). Because of the very high NK cell purity of the bioreactor expanded products, only a small population (<5%) of mature CD14^+^ and/or CD15^+^ monocytic cells could be detected, but no CD34^+^ cells, CD3^+^ T cells or CD19^+^ B cells were found (data not shown). Furthermore, we compared the expression of various NK cell specific surface antigens and NK cell function in degranulation assays on static bag and bioreactor generated NK cell products (Figure S1). The bioreactor cultures show a higher expression of activating receptors such as CD314 (NKG2D) and NCRs (i.e. CD337, CD336, CD335) (Figure S1 A). This correlated with a higher degranulation of 27% of bioreactor-expanded NK cells towards K562 compared to 14–18% for NK cells from static bag cultures (Figure S1 B). These data demonstrate that the combination of static bag cultures for progenitor cell expansion followed by efficient NK production in bioreactor systems result in a efficient production of pure NK cell products for adoptive immunotherapy trials.

**Table 3:** Overview of the quantity and quality of final UCB-NK products generated from enriched CD34^+^ cells using static bags and single use bioreactors.

	Donor	CD34^+^ cells (x10^6^)	fold expansion	CD56^+^ cells (%)	CD56^+^ cells (x10^9^)
**static bag**	**7**	1.7	1,770	63	1.9
	**8**	1.4	759	80	0.9
	**9**	1.3	1,291	70	1.2
**bioreactor**	**10**	0.9	2,549	95	2.2
	**13**	1.5	1,764	90	2.4
	**15**	1.5	2,657	92	3.7
	**16**	1.2	1,435	92	1.6

The table summarizes the generation of UCB-NK cell therapy products generated in static bags (Donor 7, 8 and 9) or bioreactors cultures (Donor 10, 13, 15 and 16).

### The effect of washing on recovery, phenotype and function of expanded NK cells

**Figure 2:**
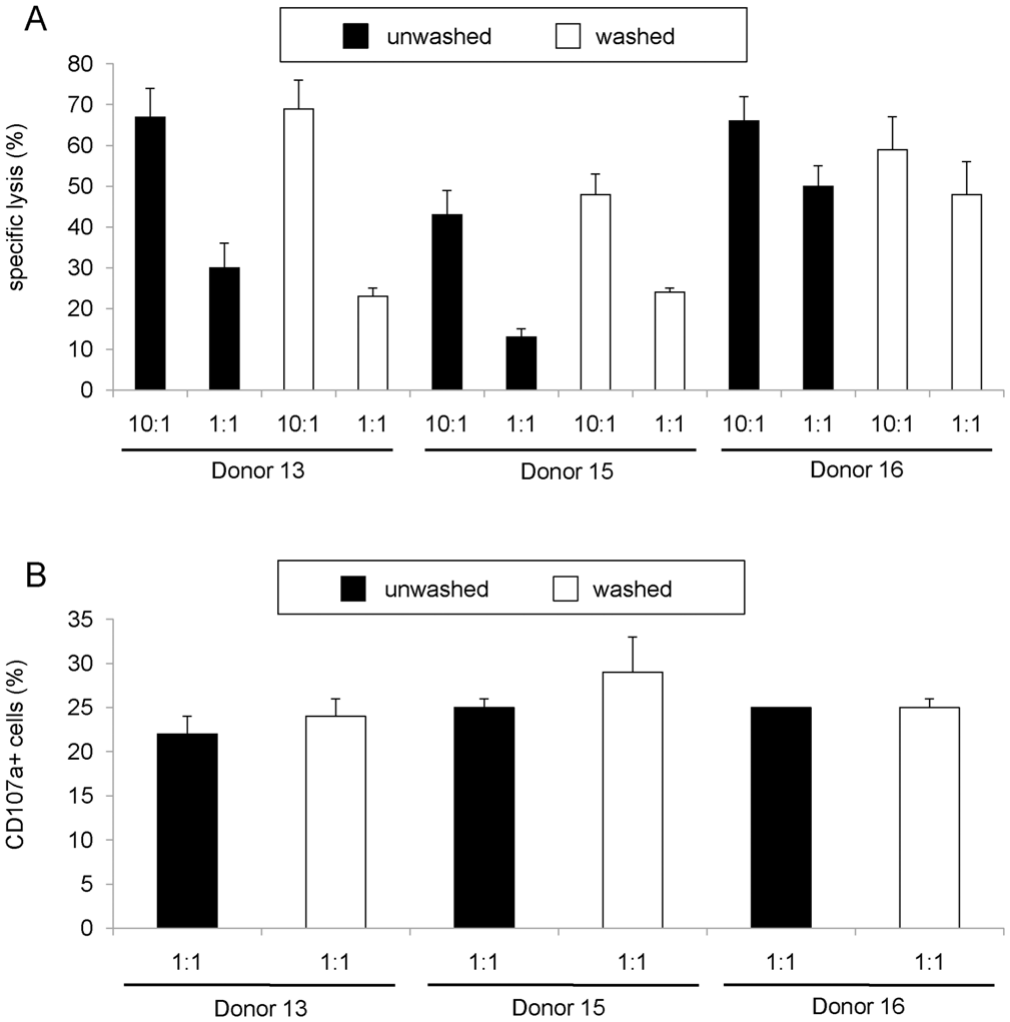
Functional activity of e*x vivo* bioreactor-expanded NK cells before and after the washing process. (**A**) Cytotoxicity of *ex vivo*-generated NK cells against K562 cells was analyzed after 18 hours of co-culture with unwashed (black bars) and washed (white bars) NK cells from three different donors at an E:T ratio of 1:1 or 10:1. (**B**) Degranulation of *ex vivo*-generated NK cells against K562 was analyzed by CD107a expression after 18 hours of co-culture after of unwashed (black bars) and washed (white bars) NK cells from three different donors at an E:T ratio of 1:1.

**Figure 3:**
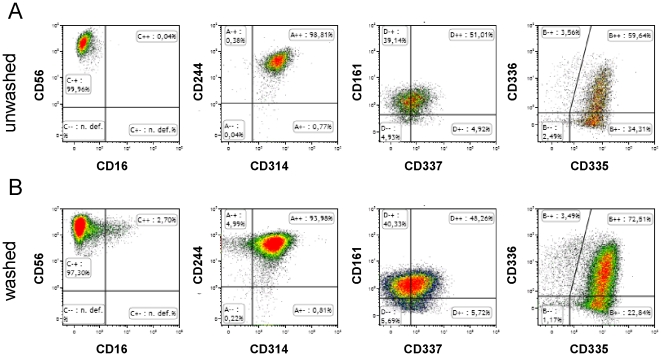
Flow cytometry analysis of e*x vivo* bioreactor-expanded NK cells before and after washing. The CD56^+^CD3^−^ lymphocytes were analyzed of unwashed (**A**) and washed (**B**) NK cell products. A representative example out of three different NK cell products is shown.

After showing that CD34^+^ UCB cells could be efficiently enriched from frozen cord blood and successfully cultured into a pure NK cells product using a closed cell culture process, we optimized downstream processing using a closed system washing procedure. Therefore, final NK cell batches were washed two times with 0.5 liter CliniMACS buffer containing 0.5% HSA in transfer bags. After this washing procedure the total cell culture volume of 1 liter was reduced and exchanged to 150 ml infusion buffer. The final dilution factor of this washing procedure was between 629–1,008 fold (n = 3), with a recovery of 82%±5% CD56^+^CD3^−^7AAD^−^ NK cells (n = 3). Cytotoxicity and CD107a-based degranulation assays using K562 as target cells showed that the cytolytic activity of the NK cell product before and after washing was not affected (Figure 2A and B). Moreover, washing of the expanded NK cells did not negatively influence the high expression of the activating receptors NKG2D (CD314), NKR-P1 (CD161), 2B4 (CD244), NKp30 (CD337), NKp46 (CD335) and NKp44 (CD336) (Figure 3). These results demonstrate that CD34^+^ UCB-derived NK cells (UCB-NK) for immunotherapy could be efficiently washed using a closed process without loss of functional and phenotypical characteristics of the bioreactor-expanded NK cells.

### UCB-NK cell therapy products fulfill specific release, biosafety and stability tests

During the validation runs of our closed culture and washing process, we monitored purity, cell numbers, viability, phenotype, activity and recovery of the UCB-NK cell products. All four validation runs in the bioreactor resulted in a final cell product containing >90% viable CD56^+^CD3^−^7AAD^−^ NK cells, and CD3^+^ T cells could not be detected (<0.01%). Importantly, cytogenetic analysis showed that the NK cell products displayed a normal karyotype. In addition, extensive testing was performed to ensure that our process was free of bacterial, fungal, mycoplasma and endotoxin contamination (Table 4). These tests were performed at the end of the NK cell production and after the washing procedure, and were negative or below specifications in all validation runs. The presence of residual SCF, IL-7, IL-15 and IL-2, which were used in the NK cell differentiation medium, was tested by specific ELISA. After washing the NK cell products, the cytokine concentrations were below the specified range of <25 pg/ml SCF, IL-7 and IL-15 and <1 U/ml IL-2.

**Table 4:** Product release testing criteria and results of the final NK cell products.

Test	Method	Specification	Donor 10	Donor 13	Donor 15	Donor 16
**NK cell number**	FCM	CD56^+^CD3^−^ NK cells	2.2×10^9^	2.4×10^9^	3.7×10^9^	1.6×10^9^
**Purity**	FCM	>70% CD56^+^CD3^−^ NK cells	95%	90%	92%	92%
**Viability**	FCM	>70% 7-AAD negative	n.a.	98%	97%	93%
**Phenotype**	FCM	>30% positivity for CD56, CD94, NKG2A, NCR and NKG2D.	yes	yes	yes	yes
**Karyotyping**	Cell culture	Normal karyotype	yes	yes	yes	yes
**Recovery**	FCM	% CD56^+^CD3^−^ NK 7-AAD negative cells.	n.a.	83%	86%	76%
**Content CD3^+^ T-cells**	FCM	<1×10^4^ CD3^+^ T cells/kg body weight of the patient	n.d.	n.d.	n.d.	n.d.
**Content CD19^+^ B-cells**	FCM	<1×10^4^ CD19^+^ B cells/kg body weight of the patient	n.a.	n.a.	n.a.	n.a.
**Sterility**	Culture	Negative for bacterial and fungal contamination	negative	negative	negative	negative
**Mycoplasm**	Luminescence assay	Negative for mycoplasm contamination	negative	negative	negative	negative
**Endotoxin**	LAL assay	<0.25 EU/ml	0.08	0.02	0.01	0.01
**Absence of cytokines**	ELISA	<25 pg/ml IL-2, IL-7, IL-15 and SCF.	yes	yes	yes	yes

The table shows an overview of product release tests and product specifications for the *ex-vivo* generated NK cells using a closed cell culture process. The table summarized the facts needed to provide a certificate of analysis to release an UCB-NK cell therapy product for a patient. n.a.  =  not analyzed in validation runs but these parameters will be scored for the clinical production and the certificate of analysis. n.d.: not detected; the test do not show any positive events. yes  =  the results of the test fulfill the specification relevant for the certificate of analysis.

**Figure 4:**
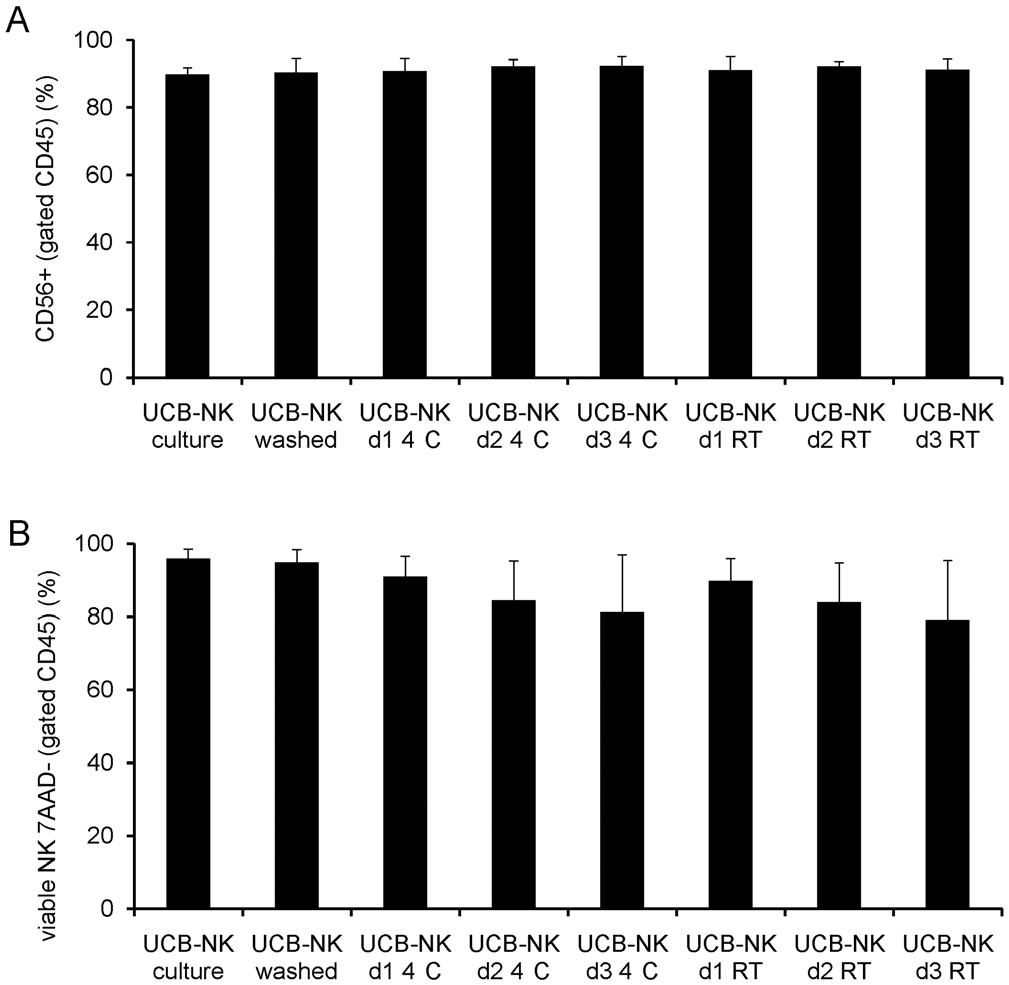
Stability tests of *ex-vivo* generated and processed NK cell products. (**A**) The NK cell content of the processed final product was followed over time, while the products were either stored at 4°C or room temperature (RT) for a maximum of 3 days. The percentage of the CD45^+^/CD56^+^ cells is displayed from 3 different stability tests. (**B**) Viability of the final NK cell product was followed over time, while the products were either stored at 4°C or room temperature (RT). The percentage of the CD45^+^/CD56^+^/7-AAD^−^ cells is displayed from 3 different stability tests.

Since we intend in our phase I clinical trial to infuse freshly prepared NK cell products without cryopreservation, we determined the stability of the NK cells in order to establish a time frame for the product release testing to be finished. Therefore, we stored UCB-NK cell products in infusion buffer (i.e. 0.9% NaCl plus 5% HSA) at 4°C or RT, and tested purity and viability at 24, 48 and 72 hours. We did not detect a decrease in purity of the NK cell product over time and also detected no differences between storage at 4°C or RT (Figure 4A). Only a small decline in viability of CD56^+^7AAD^−^ NK cells was observed at day 2 or 3 after storage at both 4°C and RT (Figure 4B). A more detailed view on the stability tests regarding the expression of several inhibitory and activating NK cell receptors showed a stable percentage of CD159a (NKG2A) and CD314 (NKG2D) positive NK cells, but the expression of the NCRs CD337, CD336, CD335 declines over time irrespective of storage at 4°C or RT (Figure S2 A–D). Our specification for NK cell infusion requires a minimum of 70% viability, and therefore we have set our expiration time for UCB-NK cells at 24 hours after final formulation.

Collectively, these results demonstrate the feasibility to generate highly pure, safe and active UCB-NK cell therapy products using a fully closed cell culture and downstream manufacturing process for evaluating in a phase I dose escalation trial in poor-prognosis patients with AML.

## Discussion

To date only a few trials have investigated adoptive NK cell infusions in patients with cancer primarily due to difficulties in isolating high numbers of NK cells from regular leukapheresis products [6]–[11]. Furthermore, *ex vivo* expansion protocols still deal with technical disadvantages by using supportive feeder cell lines that could lead to regulatory problems producing NK cell products for large-scale and multi-center trials [12]–[16]. Here, we report the successful translation of our recently developed, highly efficient cell culture protocol for the *ex vivo* generation of functional NK cell products from UCB-derived hematopoietic stem and precursor cells into a clinical applicable GMP procedure. Previously, we described a highly potent culture method for the *ex vivo* generation of fully active NK cells efficiently targeting AML and melanoma cells [18]. This cytokine-based, feeder cell-free culture process uses only human or human recombinant proteins. To translate this NK cell bioprocess into a GMP compliant procedure, we first optimized clinical-grade enrichment of CD34^+^ cells from cryopreserved UCB. Thereafter, we validated *ex vivo* generation of UCB-NK cell therapy products using a closed production process optimized for NK cell differentiation using bioreactors.

A number of studies reported previously about closed system immunomagnetic beads selection of CD34^+^ cells from cryopreserved UCB [21]–[24]. Most of these studies used the Isolex300i or the CliniMACS system, but currently only the CliniMACS system is still available for clinical application. Due to technical modifications such as different tubing sets for the CliniMACS (*i.e.* tubing 150 in older studies and tubing 161-01 in our study) and differences in the cord blood processing prior to cryopreservation one may expect variations for the efficiency of the selection procedure. For instance, the influence of different methods of volume reduction and removal of red blood cells on the recovery of CD34^+^ UCB cells has been widely studied [25]–[28]. We used the well-established EloHAES® separation method in our study [29]. Previous studies using the CliniMACS device showed a median recovery of 31% UCB CD34^+^ cells (n = 10) [21], [24]. We obtained a higher overall median CD34^+^ UCB cell recovery of 50% (n = 16), which is most likely due to the use of a highly efficient thawing procedure providing a more optimal starting cell product for CD34^+^ cell selection compared to previous studies. Querol et al. [22] used a similar thawing procedure with Pulmozyme on HES-treated cord blood units, however they used the Isolex-300-SA for CD34 selection. But they used a similar cord blood cohort, consisting of 1.11±0.5×10^9^ nucleated cells (NCs) and 3.64±2.54×10^6^ CD34^+^ cells, compared to ours with 1.08±0.4×10^9^ NCs and 3.78±1.95×10^6^ CD34^+^ cells. Using an up-to-date CliniMACS system, we isolated CD34^+^ UCB cell numbers of 1.96±1.27×10^6^ cells with a purity of 67%±14% and a recovery of 53%±15 from the used UCB cohort. These results are very similar to those of Querol et al. [22], which is 1.94±1.55×10^6^ CD34^+^ cells with a purity of 69%±16% and a recovery of 52%±12%. These findings illustrate that our current thawing and CD34^+^ selection procedures provides excellent preparation of a CD34^+^ UCB cell product for direct use or graft manipulation.

After optimizing CD34 enrichment from cryopreserved UCB, we investigated whether CliniMACS-enriched CD34^+^ UCB cells could be efficiently expanded and further differentiated into the NK cell lineage using a closed cell culture system. Recently, Sutlu et al. reported that large amounts of highly active NK cells can be produced from peripheral blood mononuclear cells (PBMCs) in a closed, automated, bioreactor under feeder-free conditions [17]. Interestingly, bioreactor cultures yielded a final product containing a clinically relevant NK cell dose (mean 9.8×10^8^ NK cells). Moreover, they observed that NK cells expanded in a bioreactor, compared to flasks and bags, displayed higher cytotoxic activity possibly attributed to a higher expression level of NKp44. In agreement with the studies of Sutlu et al., we also obtained a much better purity, functionality and significant better NK cell expansion using the Wave™ or Biostat™ bioreactor systems during the NK cell differentiation process. For efficient NK cell generation it seems, that an optimal gas exchange for the cell suspension could be provided by rocking or waving of the cell suspension. Finally, we performed safety and release tests on the NK cell end products (summarized in Table 4). The genetic stability was analyzed by karyotype analysis and all NK cell products had a normal karyotype. Furthermore, all generated products were negative for bacterial, fungal or mycoplasm contamination. After washing of the product, the volume was reduced to 150 ml prior infusion. Cytokine levels were efficiently reduced to below 25 pg/ml. Immunophenotyping analysis demonstrated high purity, viability and activated phenotype of the NK cell product with the complete absence of CD3^+^ T cells. We additionally demonstrate, that the cell culture process is safe and that the product could be further processed, stored and safely released for patients.

In conclusion, we successfully adapted our method into a closed-system bioprocess for production of allogeneic NK cell batches under GMP conditions, in order to utilize *ex vivo*-expanded NK cells for adoptive immunotherapy in poor-prognosis AML patients. Large-scale experiments using gas-permeable culture bags and up-scaling of the NK cell expansion step into the bioreactor systems resulted in the generation of products containing more than 3.5×10^9^ NK cells with a purity of up to 95%. Furthermore, the UCB-NK-cell products could be finally processed for infusion using a closed system and be stored until all product control tests will be available in order to release the UCB-NK-cell therapy product. The safety and toxicity of infusing these allogeneic *ex vivo*-expanded UCB-NK cell therapy products will be investigated in phase I dose escalation trial in elderly AML patients not eligible for allogeneic stem cell transplantation.

## Materials and Methods

### Cell lines

Cell line K562 (LGC Standards, Wesel, Germany) was cultured in Iscove's modified Dulbecco's medium (IMDM; Invitrogen, Carlsbad CA, USA) containing 50 U/ml penicillin, 50 µg/ml streptomycin and 10% fetal calf serum (FCS; Integro, Zaandam, the Netherlands).

### Isolation of CD34^+^ stem and progenitor cells

UCB units have been obtained at birth after normal full-term delivery after written informed consent with regard of scientific use from the cord blood bank of the Radboud University Nijmegen Medical Center (RUNMC, Nijmegen, The Netherlands). The use of these UCB units for our study was approved by the RUNMC Institutional Review board. UCB samples were stored at room temperature and processed within 24 h after collection. Before storage, the red blood cell content has been reduced using standard EloHAES® separation and the mononuclear cells have been washed, cryopreserved and stored in liquid nitrogen [29]. Stored UCB units were thawed at 37°C and resuspended in thawing buffer consisting of CliniMACS PBS/EDTA buffer (Miltenyi Biotech, Bergisch Gladbach, Germany), 5% HSA (Sanquin blood bank, Amsterdam, The Netherlands), 3.5 mM MgCl_2_ (Pharmacy Department, RUNMC, Nijmegen, The Netherlands) and 100 U/ml Pulmozyme (Roche, Almere, The Netherlands). Thawed UCB cells were incubated for 30 minutes at room temperature (RT) and subsequently centrifugated. After two washing steps, thawed UCB cells were resuspended in 8 ml washing buffer consisting of CliniMACS PBS/EDTA buffer, 0.5% HSA, 3.5 mM MgCl_2_ and 100 U/ml Pulmozyme and labeled for 30 minutes at RT with 0.75 ml CliniMACS CD34 reagent (Miltenyi Biotech) and 1 ml Nanogam (Sanquin blood bank, Amsterdam, The Netherlands). After incubation, the CD34-labeled UCB sample was washed and resuspended in 100 ml washing buffer. The automated CliniMACS cell separator was equipped with a closed disposable CliniMACS tubing set type 161-01 (Miltenyi Biotech). The CD34^+^ cell selection was performed using an automated program and after the enrichment procedure, the CD34^+^ cell fraction was collected, and the cell number and purity were analyzed by flow cytometry. Finally, the obtained CD34^+^ UCB cells were used directly for the NK cell generation bioprocess.

### *Ex vivo* expansion and differentiation of CD34-positive progenitor cells

CD34^+^ UCB cells were transferred into Vuelife™ bags 290AC or 750AC (Cellgenix, Freiburg, Germany), and expanded and differentiated according to culture method III as described previously with some minor modifications [18]. In brief, for day 0–9 Expansion Medium I was used consisting of Glycostem Basal Growth Medium (GBGM®) for cord blood (CB) (Clear Cell Technologies, Beernem, Belgium) supplemented with 10% human serum (HS; Sanquin Bloodbank, Nijmegen, The Netherlands), a high-dose cytokine cocktail containing SCF, Flt3L, TPO and IL-7 (all CellGenix, Freiburg, Germany) and a low-dose cytokine cocktail consisting of GM-CSF (Neupogen) (Amgen, Breda, The Netherlands), G-CSF and IL-6 (both CellGenix, Freiburg, Germany). Between day 10 and 14, Expansion II medium was used in which TPO was replaced by IL-15 (CellGenix, Freiburg, Germany). During the first 14 days of culture, low molecular weight heparin (LMWH) (Clivarin®; Abbott, Wiesbaden, Germany) was added to the expansion medium. Cell cultures were refreshed with new medium every 2–3 days, and adjusted to a cell density of >0.5×10^6^/ml. Cultures were maintained in a 37°C, 95% humidity, 5% CO_2_ incubator. Expanded cultures in Vuelife™ bags were either maintained in Vuelife™ bags or transferred to a bioreactor at around day 14. We have used both the single use WAVE Bioreactor™ System 2/10 (GE Health, Uppsala, Sweden) and BIOSTAT® CultiBag RM system (Sartorius Stedim Biotech, Göttingen, Germany). From day 14 onward, expanded CD34^+^ UCB cells were differentiated and further expanded using NK cell differentiation medium. This medium consisted of Glycostem Basal Growth Medium (GBGM®) for cord blood (CB) as used for the CD34 expansion step supplemented with 10% HS, the low-dose cytokine cocktail (as previously mentioned) and a new high-dose cytokine cocktail consisting of IL-7, SCF, IL-15 and IL-2 (Proleukin®; Chiron, München, Germany). The cell density was checked two times a week and adjusted to ∼1.0×10^6^ cells/ml by the addition of GBGM® NK cell differentiation medium. The conditions of the bioreactor were as follows: temperature 37°C, CO_2_ 5%, airflow 0.1–0.2 l/min, rocking rate 10/min, rocking angle of 6°.

### Flow cytometry

Cell numbers and expression of cell-surface markers were determined by flow cytometry. For immunophenotypical staining, cells were incubated with the appropriate concentration of antibodies for 30 min at 4°C. After washing, cells were resuspended in Coulter® Isoton® II Diluent (Beckman Coulter) and analyzed using the Coulter FC500 flow cytometer (Beckman Coulter). For determining the content of CD34^+^ cells in the UCB and the purity of the CD34 selected cells the following monoclonal antibodies were used: CD45-FITC (J33) and CD34-PE (581) (both from Beckman Coulter, Woerden, The Netherlands). The population of living CD34^+^ cells was determined by exclusion of 7AAD (Sigma, Bornem, Belgium) positive cells. Analysis was performed according to the most actual ISHAGE protocol. The purity of the end product after washing was determined using the following monoclonal antibodies were used: CD3-FITC (UCHT1) (Beckman Coulter, Woerden, The Netherlands); CD56-PE (NCAM16-2) (BD Biosciences Pharmingen, Breda, The Netherlands), anti-CD45-ECD (J33) (Beckman Coulter, Woerden, The Netherlands). The population of living CD56^+^ cells was determined by exclusion of 7AAD (Sigma) positive cells. Ten color analysis was used to determine the phenotype of the cultured NK cells. The following monoclonal antibodies were used in the appropriate concentration: CD16-FITC (NKP15), CD336(NKp44)-PE (Z231), CD3-ECD (UCHT1), CD337(NKp30)-PC5.5 (Z25), CD335(NKp46)-PE-Cy7 (BAB281), CD314(NKG2D)-APC (ON72), CD244(2B4)-APC-alexa700 (C1.7.1), CD56-APC-Alexa750 (N901), CD161-PB (191B8), CD45-PO (J33) (all provided by Beckman Coulter, Marseille, France). The acquisition was performed on the Navios™ flowcytometer and the data were further analyzed using the Kaluza™ software (all from Beckman Coulter, Miami, Florida, USA).

### Flow cytometry-based cytotoxicity and degranulation studies

Flow cytometry-based cytotoxicity assays were performed as described previously [18], [30]. Briefly, after incubation for 4 h or overnight at 37°C, 50 µl supernatant was collected and stored at −20°C for later use to measure cytokine production. Cells in the remaining volume were harvested and the number of viable target cells was quantified by flow cytometry. Target cell survival was calculated as follows: % survival  =  {[absolute no. viable CFSE^+^ target cells co-cultured with NK cells]/[absolute no. viable CFSE^+^ target cells cultured in medium]}*100%. The percentage specific lysis was calculated as follows: % lysis  =  {100-[% survival]}. Degranulation of NK cells during co-culture was measured by cell surface expression of CD107a [31]. After 18 hrs of incubation at 37°C, the percentage of CD107a^+^ cells was determined by flow cytometry.

### Preparation of the final NK cell product

At the end of culture, NK cells were harvested, and the number and viability of CD56^+^ cells was determined by flow cytometry and ACT counter (Beckman Coulter). The UCB-NK-cell product was transferred into 600 ml transfer bags (Baxter, Deerfield, USA), centrifugated 200 g for 15 min without break and the supernatant was collected for testing of bacterial, fungal and mycoplasm contamination. NK cells were resuspended and washed twice with 500 ml CliniMACS PBS/EDTA buffer supplemented with 0.5% HSA (Sanquin Blood Bank, Amsterdam, The Netherlands). After washing, NK cells were resuspended in infusion buffer consisting of NaCl 0.9% + 5% HSA. Finally, viable number of CD56^+^CD3^−^ NK cells in the end-product was determined by flow cytometry and the concentration of residual SCF, IL-7, IL-15 and IL-2 was measured by ELISA (R&D Systems, Abingdon, Oxon, UK).

### Karyotyping of the NK cell product

Cytogenetic analysis was performed on the final NK cell products according to standard methods. In total 20 metaphases were G-banded using trypsin and Giemsa (GTG) and were examined per case. Karyotypes were described according to ISCN 2009 [32].

### Sterility testing of the NK cell product

Before and after washing in bags, samples were taken and processed to check for bacterial and fungal contaminations. These samples were transferred to Bactec Ped plus flasks (BD). Bacterial growth till day 6 should be reported as positive. The testing was done by the Department of Microbiology, RUNMC, Nijmegen, The Netherlands.

### Mycoplasma testing

Mycoplasma detection was performed on final products using the MycoAlert® Mycoplasma detection kit (Lonza, Rockland, USA) following the manufacturer's instructions. The signals were measured with the Fluostar Optima (BMG Labtech, IJsselstein, The Netherlands).

### Endotoxin test

Endotoxin level in the final products was determined using the chromogenic Limulus Amebocyte Lysate (LAL) assay (Charles River Endosafe, Charleston, SC, USA) following the manufacturer's guidelines by the Pharmacy Department, RUNMC, Nijmegen, The Netherlands. A level of <0.25 EU/ml was set as negative endotoxin limit.

### Cytokine detection

Cytokine levels in the final products were determined using ELISA. Briefly, Maxisorp 96-well plates (NUNC) were coated overnight with 1 µg/ml of monoclonal coating antibody for IL-2, IL-7, IL-15 and SCF (all from R&D systems, Abingdon, Oxon, UK). For sample detection, biotinylated antibodies were added for IL-2 (0.2 µg/ml polyclonal Ab), IL-7 (0.2 µg/ml polyclonal Ab), IL-15 (0.25 µg/ml monoclonal Ab) and SCF (0.05 µg/ml polyclonal Ab), respectively. The extinction was measured by the TiterTek Multiscan MCC/340 plate reader (Titertek, Huntsville, AL). Concentrations of triplicate measurements were determined using a standard curve ranging between 1 to 2000 pg/ml of the specific cytokine.

### Statistics

Results from different experiments are described as mean ± standard deviation of the mean (SD), range and median. Statistical analysis was performed using students t-test. A p-value of <0.05 was considered statistically significant.

## Supporting Information

Figure S1 **Flow cytometry analysis for phenotype and function of e** ***x vivo*** **expanded NK cells using static bags or bioreactor cultures.** (**A**) The CD45^+^CD56^+^CD3^−^ lymphocytes were analyzed for several NK cell specific surface antigens for bioreactor cultures (black bars) and static bags (grey bars). (**B**) NK cell functionality was tested in a CD107a degranulation assay and the percentage of degranulating cells (CD107^+^) was analyzed. Bioreactors cultures from donor 10 and 13 were compared with static bag cultures from donor 7 and 9 in an overnight co-culture with an E:T ratio of 1∶1.
(TIF)

Figure S2 **Stability tests of** ***ex-vivo*** **generated and processed NK cell products.** (A–D) The CD45^+^CD56^+^CD3^−^7AAD^−^ lymphocytes from two different donors (A&B and C&D) were analyzed for several NK cell specific surface antigens and followed over time. (A and C) The products were either stored at 4°C or (B and D) at room temperature (RT) for a maximum of 3 days.
(TIF)
