# Supplementary figures and images for: Clinical-Grade Generation of Active NK Cells from Cord Blood Hematopoietic Progenitor Cells for Immunotherapy Using a Closed-System Culture Process
Source: PLoS One. 2011 Jun 16;6(6):e20740. doi: 10.1371/journal.pone.0020740 (PMC3116834; doi:10.1371/journal.pone.0020740)

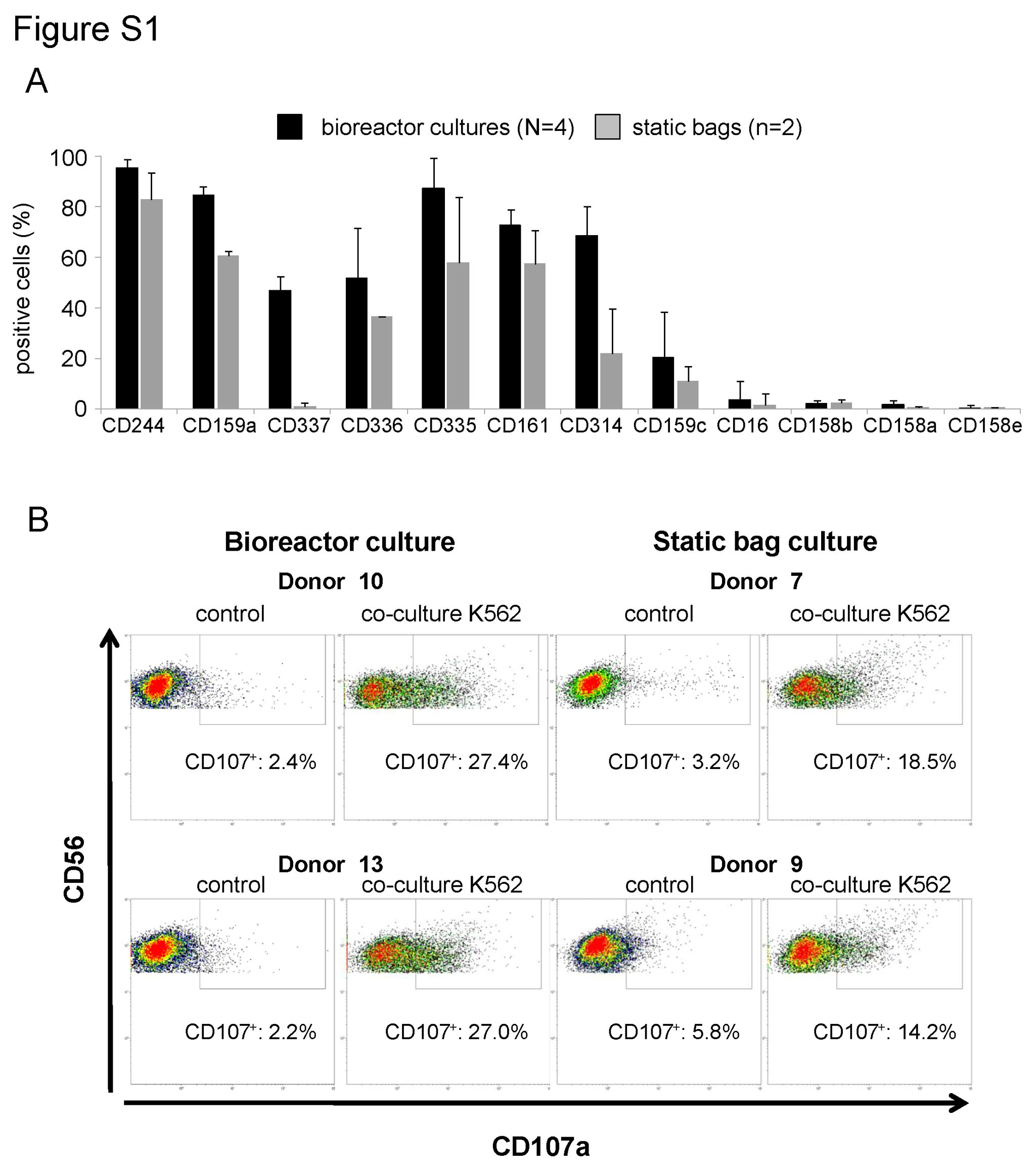

Supplement: Figure S1 — Flow cytometry analysis for phenotype and function of e x vivo expanded NK cells using static bags or bioreactor cultures. (A) The CD45+CD56+CD3− lymphocytes were analyzed for several NK cell specific surface antigens for bioreactor cultures (black bars) and static bags (grey bars). (B) NK cell functionality was tested in a CD107a degranulation assay and the percentage of degranulating cells (CD107+) was analyzed. Bioreactors cultures from donor 10 and 13 were compared with static bag cultures from donor 7 and 9 in an overnight co-culture with an E:T ratio of 1∶1. (TIF) [file pone.0020740.s001.tif]

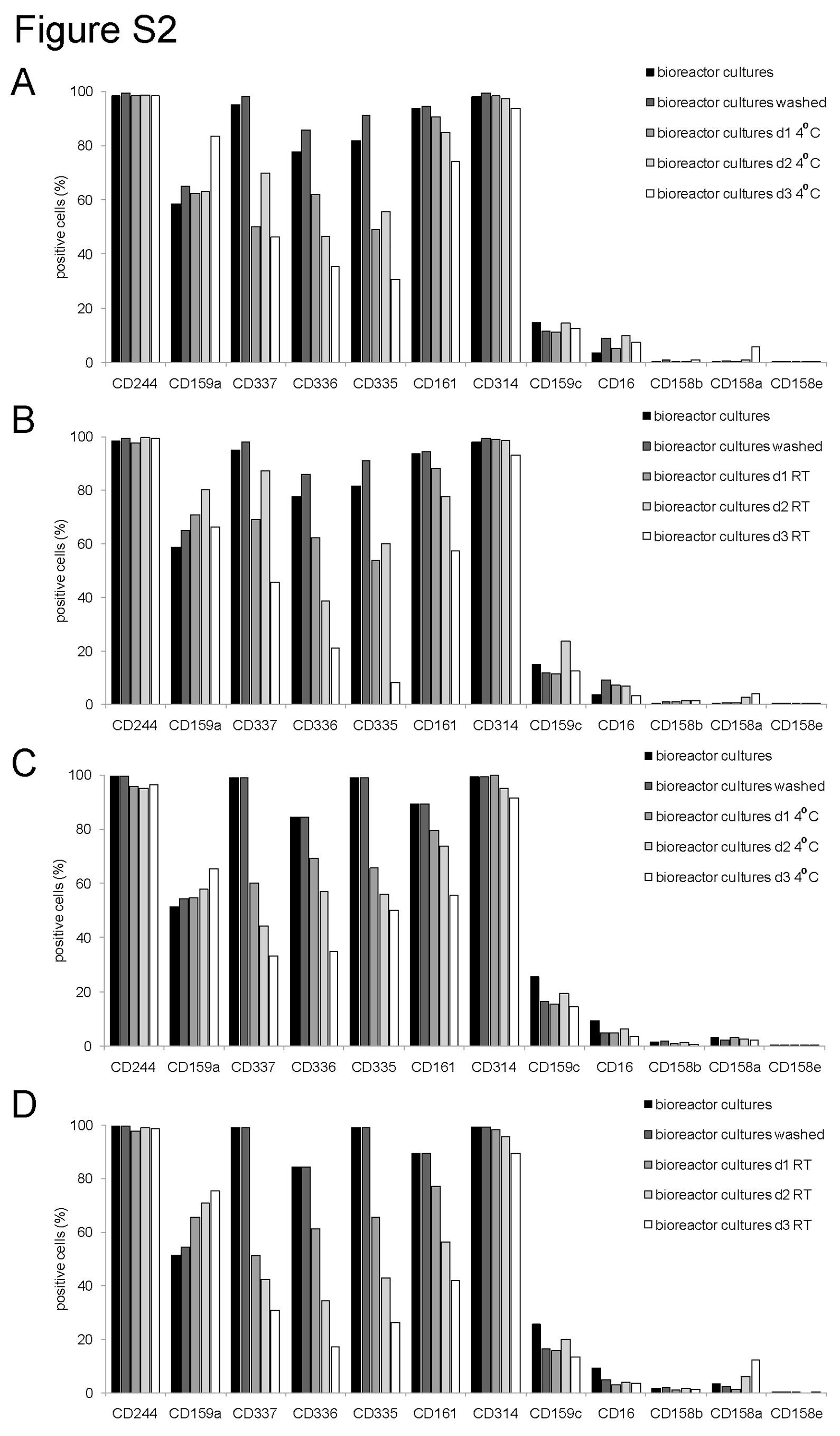

Supplement: Figure S2 — Stability tests of ex-vivo generated and processed NK cell products. (A–D) The CD45+CD56+CD3−7AAD− lymphocytes from two different donors (A&B and C&D) were analyzed for several NK cell specific surface antigens and followed over time. (A and C) The products were either stored at 4°C or (B and D) at room temperature (RT) for a maximum of 3 days. (TIF) [file pone.0020740.s002.tif]
